# Spatial Interpolation of Fine Particulate Matter Concentrations Using the Shortest Wind-Field Path Distance

**DOI:** 10.1371/journal.pone.0096111

**Published:** 2014-05-05

**Authors:** Longxiang Li, Jianhua Gong, Jieping Zhou

**Affiliations:** 1 Institute of Remote Sensing and Digital Earth, Chinese Academy of Sciences, Olympic Science & Technology Park of CAS, Beijing, China; 2 Institute of Remote Sensing and Digital Earth, Chinese Academy of Sciences, Olympic Science & Technology Park of CAS, Beijing, China and Zhejiang-CAS Application Center for Geoinformatics, Jiashan, Zhejiang, China; The Ohio State University, United States of America

## Abstract

Effective assessments of air-pollution exposure depend on the ability to accurately predict pollutant concentrations at unmonitored locations, which can be achieved through spatial interpolation. However, most interpolation approaches currently in use are based on the Euclidean distance, which cannot account for the complex nonlinear features displayed by air-pollution distributions in the wind-field. In this study, an interpolation method based on the shortest path distance is developed to characterize the impact of complex urban wind-field on the distribution of the particulate matter concentration. In this method, the wind-field is incorporated by first interpolating the observed wind-field from a meteorological-station network, then using this continuous wind-field to construct a cost surface based on Gaussian dispersion model and calculating the shortest wind-field path distances between locations, and finally replacing the Euclidean distances typically used in Inverse Distance Weighting (IDW) with the shortest wind-field path distances. This proposed methodology is used to generate daily and hourly estimation surfaces for the particulate matter concentration in the urban area of Beijing in May 2013. This study demonstrates that wind-fields can be incorporated into an interpolation framework using the shortest wind-field path distance, which leads to a remarkable improvement in both the prediction accuracy and the visual reproduction of the *wind-flow effect*, both of which are of great importance for the assessment of the effects of pollutants on human health.

## Introduction

Public health studies of air-pollution exposure require accurate predictions of concentrations at unmonitored locations to minimize the misclassification of exposure levels [1]. Recent studies have reported that intra-urban-scale variations in air-pollution concentration may exceed the differences between cities [2], [3], suggesting the potential importance of predicting air-pollution at fine spatial scales [4], [5]. Correspondingly, estimates of exposure to pollutants on small temporal scales are necessary to study the short-term or acute impacts of air-pollution [6]. Spatial interpolation models, landuse regression (LUR) models, remote-sensing-based models and diffusion models are robust tools for intra-urban air-pollution prediction [7], [8]. However, spatial interpolation techniques, which generate concentration surfaces from *in situ* observations, are preferred for the estimation of real-time concentrations when data availability and software and hardware costs are taken into account [3], [9], [10].

Wind is a key meteorological factor that has major impacts on the movement and distribution of air pollutants in a region. When the wind-speed is relatively high, local wind-field exert substantial influence on the horizontal transport of air-pollution; this phenomenon is known as the *wind-flow effect* [11], [12]. For example, areas downwind of highways are more heavily exposed to traffic-related pollutants than are upwind areas. This effect illustrates the necessity of incorporating wind-field into spatial interpolation. In a number of recent studies, the consideration of a negative correlation between air-pollution concentration and wind-speed has led to the application of the wind-speed as an auxiliary variable in multi-variable interpolation methods [9], [13], [14]. Although there have been several attempts to incorporate long-term, large-scale wind-fields into corresponding air-pollution estimations, short-term, small-scale wind-fields have not been extensively used for this purpose, because no direct numerical relations exist between the angle of the wind-direction and the concentration level in such cases. As a result, these approaches fail to capture the expected short-term effects of the wind flow.

By including the wind-fields indirectly, some regression-based methods are able to capture the complex features of pollutant distributions [2], [3], [14]. A recent study assessed the use of the wind-direction in LUR to improve predictions of nitrogen dioxide levels in Toronto-Hamilton area [11], [15]. This method shows great potential, as it quantifies the influence of the wind-direction with the downwind distances from highways. However, real-time air-pollution assessment using this model is economically infeasible because of the cost of collecting sufficiently diverse data sets. Therefore, one objective of the present study is to incorporate wind-fields directly into interpolation frameworks.

Most interpolation techniques depend on Euclidean or straight-line distances to compute spatial dependency. However, the complex features of certain spatial phenomena impede the ability to obtain accurate dependency descriptions using Euclidean distances [16], [17]. An appropriate non-Euclidean distance may outperform the Euclidean distance in determining such types of spatial dependency and in capturing complex features [18]. The shortest path distance (SPD) is an important subclass of non-Euclidean distance and has exhibited great potential in diverse interpolation studies. The hydrological distance, a derivative of the SPD, has been used to characterize the spatial configurations, connectivity and directionality of the water temperatures and chemical pollutants in stream networks [17], [19]–[22]. Accounting for geological anisotropy has led to the interpolation of deposits in conjunction with shortest anisotropy path distances [23]. Along-road continuity has been described in carbon dioxide estimations after replacing the Euclidean distance with the SPD [24]. The inclusion of the effect of topographical factors in simulations of the genetic dispersion path has led to the development of the concept of effective distance, which is the length of a virtual movement route. Although the quantitation of these factors remains unclear, this metric exhibits a greater correlation with genetic variance than does the straight-line distance and has been used to characterize the nonlinear features of genetic dispersal [25]. Road-network connectivity has also been incorporated into the interpolations of urban travel speeds using the approximate road-network distance, another derivative of the SPD [26]. The works listed above are important references for the methodology presented in this paper. However, the characteristics of the wind-flow effect differ from those investigated in these previous works. Current SPD techniques are insufficient to successfully capture such features. To address this shortcoming, another derivation of SPD, the shortest wind-field path distance (SWPD), is proposed to determine the spatial dependency effected by a wind-field and is exploited to integrate wind-fields into interpolation frameworks.

In this study, a new interpolation method based on the SPD is developed to describe the influence of the wind-field. This technique is then applied to generate concentration surfaces for fine particulate matter of less than 2.5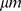 in diameter (PM_2.5_) on the experimental dates in the study area. Comparisons are performed between this technique and the conventional methodology to illustrate the improvements achieved by including the wind-field.

## Materials and Methods

### Data collection and processing

#### Study area and context

**Figure 1:**
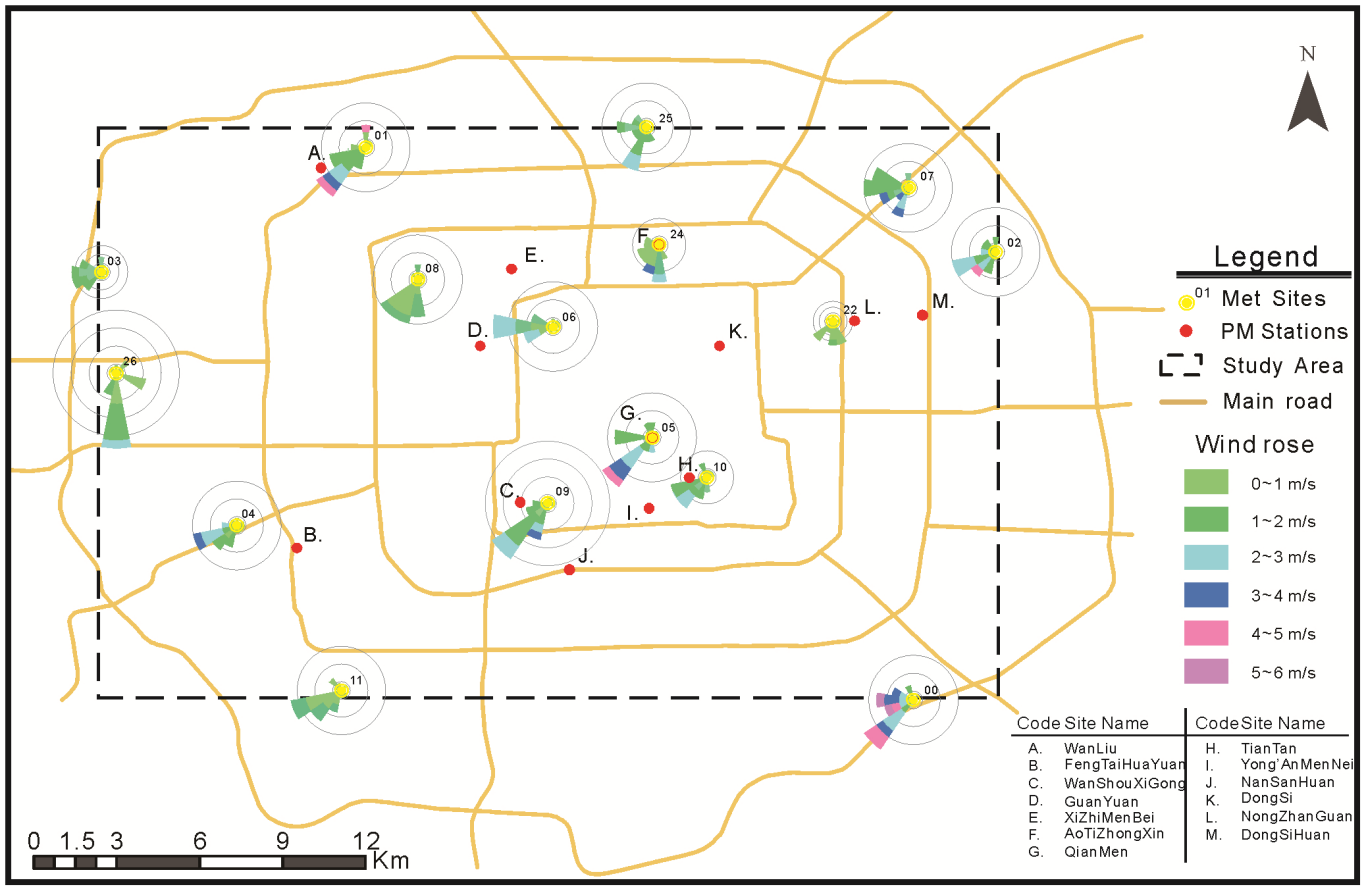
Study area and locations of PM_2.5_ monitoring stations and meteorological sites with daily wind roses for May 18^th^.

Beijing, the capital of the People's Republic of China, is an international metropolis and has experienced a rapid increase in urban population, energy consumption and vehicle numbers over the past several decades [27]. An urban area inside the surrounding ring road (Fig. 1) was selected as the study area (approximately 30×30 km) because of the relatively dense air-pollution monitoring network that is present in this area. Six non-consecutive days in May 2013 with major air-pollution in terms of PM2.5 and daily wind-speeds above 1.5 m/s were selected as the experimental dates because no accurate wind-direction measurements were available for low-speed wind conditions.

#### Observed PM2.5 concentration

To improve air-pollution monitoring, a network of 35 automated stations has been established by the Beijing Environmental Protection Bureau (BJEPB). Each station measures hourly PM_2.5_ concentrations and releases real-time data to the public through the Beijing Municipal Environmental Monitoring Center (www.bjmemc.com.cn). 13 urban sampling sites compose a dense urban monitoring subsystem across the study area (Fig. 1). This monitoring network enables the detection of real-time, small-area variations in the PM_2.5_ concentration. We collected an experimental data set from all 13 sites for six selected dates. The concentration data are given in units of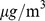. Daily average concentrations were calculated from the hourly data.

#### Observed wind-fields

Hourly wind-field observations were obtained through the Chinese Meteorological Data Service Platform (cdc.bjmb.gov.cn). The measurements were collected over a network of 16 weather stations throughout the study area operated by the Beijing Meteorological Bureau (Fig. 1). The daily average wind-speed and wind-direction were calculated from the hourly real-time data. Influenced by the complex urban morphology, the urban wind-fields exhibit dramatic small-scale variations that cannot be captured by model-simulated fields with overlarge grid sizes [11].

### Methodology

At the heart of the proposed method is the shortest-path analysis. An appropriate simulation of air-pollution movement using this method entails the construction of a cost surface, which lays the foundation for the shortest-path analysis. After the SWPD between every pair of unmonitored locations and sampling locations is obtained, this distance metric is used to determine the spatial dependency. Inverse Distance Weighting (IDW) in conjunction with SWPD is then implemented to calculate the concentration surface. Therefore, this method consists of generating a continuous wind-field, then implementing the shortest-path analysis and finally creating the estimation surface using SWPD-based IDW.

#### Generation of continuous wind-field

**Figure 2:**
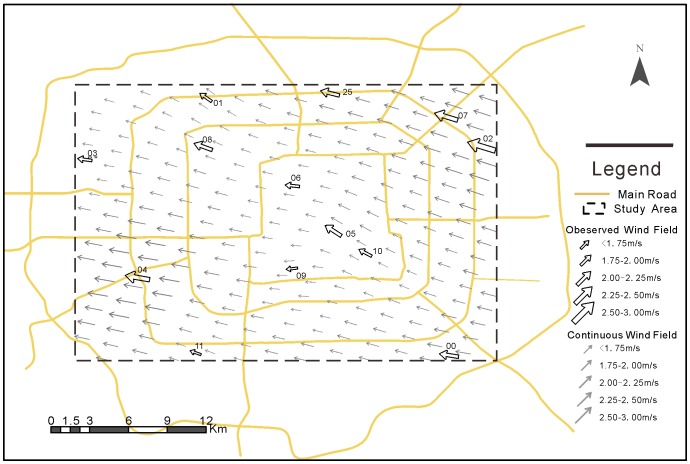
Observed wind-field and continuous wind-field generated from the monitoring data of May 28^th^.

The creation of the cost surface for shortest-path analysis first requires the generation of continuous wind-field. This process also involves spatial interpolation. Unlike other scalar weather variables, a wind-field is a vector quantity whose interpolation is unique in meteorology. Typically, one wind-vector is decomposed into two Cartesian wind components (an east-west component and a north-south component). Each component is then interpolated separately into a corresponding surface using multiquadric (MQ) radial basis functions (RBF) [28]. The wind-field is then constructed backward from the two Cartesian-component surfaces using trigonometry (Fig. 2). This methodology has been widely used to interpolate diverse vector-type data since its proposal and is considered to be a robust approach for various meteorological studies [28]. In this study, a continuous wind-field is established using a grid size of 0.5 km, which is an appropriate resolution for urban air-pollution research [29].

#### Shortest-path analysis

The shortest-path analysis includes two stages. First, a continuous wind-field is modeled onto a cost surface that depicts the movement cost between adjacent cells. Second, a shortest-path algorithm is implemented to acquire the SWPDs between locations.

#### Creating a cost surface using wind-field data

The movement of PM2.5 from one location to another may be facilitated or impeded by the local wind-field. A cost surface must be well defined based on the properties of the air-pollution movement to ensure that each shortest path acquired represents the true movement trajectories and thus reveals the path along which the two locations are related.

**Figure 3:**
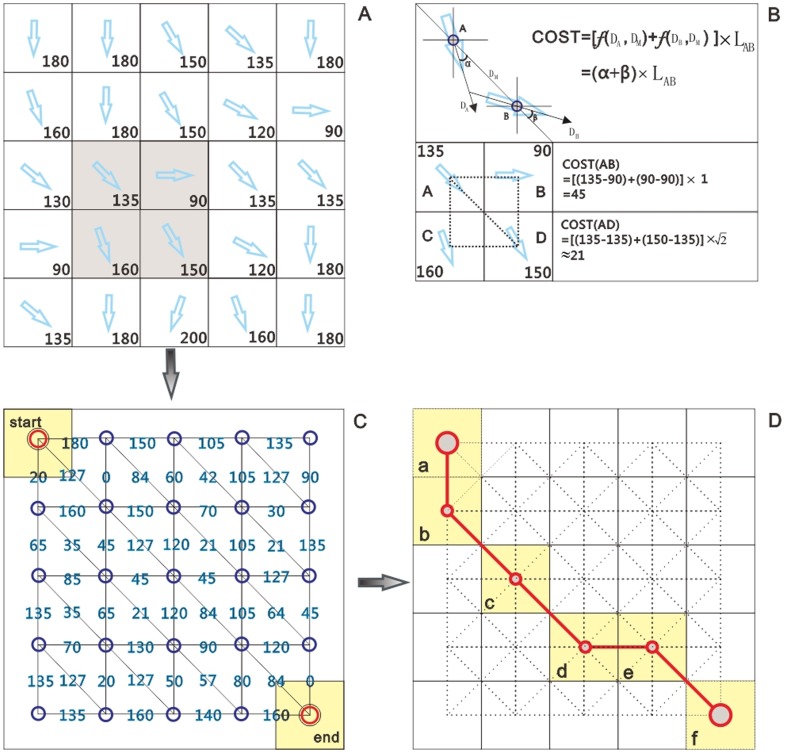
Steps for calculating SWPDs using a wind-field. (A) Grid-based representation of a wind-field. (B) Computing the cost associated with the edge between adjacent cells. (C) Reforming a grid-based wind-field into a graph. (D) SWPD calculated between starting point A and ending point F.

The grid-based representation of field data is sufficient to depict the cost of traversing each cell but is incapable of representing the movement cost associated with not only the distance between cells but also the relative positions of adjacent cells. An alternate methodology is to reform the grid raster (Fig. 3A) into a graph (Fig. 3C) with pixels as vertices and virtual connecting lines as edges, where each edge has an associated cost value that indicates the cost of traveling along this edge.

The calculation of the edge cost that depicts the movement difficulty between adjacent cells is performed based on Gaussian dispersion model, which is the standard model for the study of the transport of airborne contaminants under the influence of wind-field. This model simulates a cross section of the air-pollution dispersion and assumes that both the horizontal and vertical concentration distributions are normal [30], [31]. The basic formula for this model can be written as follows: 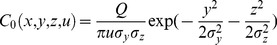
(1)where 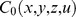 is the concentration at ground level, 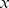 is the downwind distance, 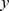 is the horizontal distance between the point of interest and the centerline, 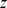 is the height of the emission source, 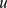 is the horizontal wind-speed, 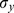 is the standard deviation of horizontal dispersion and 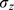 is the standard deviation of vertical dispersion.

The Cartesian coordinates (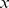 and 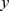) in the model can be transformed into polar coordinates (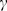 and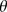) as shown below [32]:
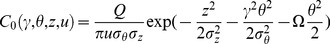
(2)where 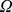 is a correction term given by
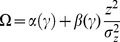
(3)with 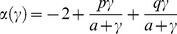
(4)




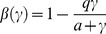
(5)where 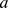, 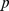
and 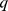
are dispersion parameters that depend on the atmospheric stability. Further information can be found in the cited references. When the focus is placed only on horizontal diffusion and downwind advection, the formula can be written as
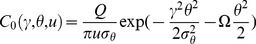
(6)which indicates that the concentration will decrease with increasing downwind distance (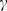) and azimuth (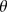), both of which are used to determine the movement cost in the following steps. However, this formula is too complicated to be directly used in calculation, and the following simplified version is applied instead:
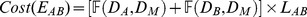
(7)where A and B are two points in the wind-field, 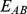
is the edge connecting these two points, 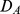 and 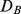 are the wind-directions at these two points and 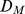 is the direction of 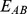, namely, the potential movement direction. 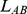 is the length of 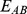, which is functionally equivalent to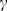. The function 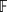 is used to calculate the azimuth which is functionally identical to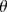. It is important to note that the Gaussian dispersion model is designed to simulate the diffusion of contaminants from definite sources and cannot be directly applied in this study because there are no stable emission sources. However, this model illustrates the origin of the movement cost, which serves as the foundation of this section.

#### Implementing shortest-path analysis

Based on the establishment of the cost surface, shortest-path algorithms can be used to calculate the paths with the minimum accumulated movement cost between point pairs, indicating the most likely path along which the two points are related (Fig. 3d). In this study, the classic Dijkstra algorithm [33] is employed. The shortest paths and their associated costs are computed as the output of this step. However, SWPD is not measured as a summation of movement costs but as the total length of the shortest path segments between location pairs.

#### Interpolation based on SWPD

After the SWPD between each pair of prediction locations and measurements is acquired, SWPD-based interpolation can be used to generate estimation surfaces. IDW in conjunction with the SWPD was selected as the technique used to incorporate wind-field in this study. Although the total sample size in this study is small, the density of the observation network permits the estimation of small-area variations in the air-pollution concentration using the proposed method [34]. The feasibility of the method is confirmed by the low mean squared error and mean absolute error in the following sections.

The IDW approach aims to predict the pollutant concentration at a given location based on a weighted average of the measurements obtained at surrounding stations. As a direct application of Tobler's First Law (TFL), the relations between the point of interest and the nearby stations are determined by the distances between them. The method takes the following form:
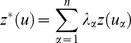
(8)where
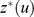
is the estimate at location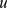, 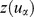
is the measurement at location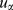, n is the number of stations used for the estimation and
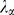
is the interpolation weight of the measurement at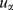. The calculation of
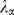
takes the following form:
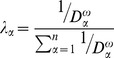
(9)where 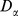
is the distance between the monitoring station numbered
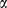
and the point of interest; 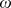 is the exponent of the distance and is set to 2 by default. The incorporation of the SWPD can be achieved by replacing the distances with the SWPD values, as demonstrated below:
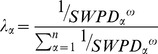
(10)where
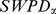
is the shortest path distance in the wind-field between the monitoring station numbered
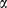
and the point of interest.

## Results

Here, comparisons are made on various temporal scales between IDW based on the SWPD (IDWS), as proposed in this paper, and IDW based on the Euclidean distance (IDWE). First, a cross-validation by “leaving one out” is performed to assess the estimation accuracy, as described below. Second, the abilities of the two methods to visually reproduce the wind-flow effect in the interpolation results are also compared. The method used in cross-validation involves temporarily removing one PM_2.5_ measurement from the data set and then predicting the concentration at this location based on the remaining measurements using the same methodology.

The three comparison criteria below are used to assess the performance of the interpolations:
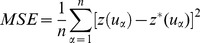
(11)




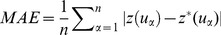
(12)




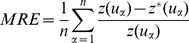
(13)The mean squared error (MSE) measures the average squared difference between the removed true PM_2.5_ measurement
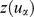
and its estimate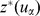. The mean absolute error (MAE) measures the average absolute difference between
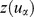
and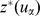. The mean relative error (MRE) measures the average relative deviation between
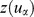
and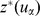. In the case of reasonably accurate estimation, the values of all three statistics should be close to zero [26], [34], [35].

### Interpolation of the daily PM_2.5_ concentration

Daily estimation surfaces for the PM_2.5_ concentration on the experimental dates were calculated using IDWS and IDWE. The local prediction is improved when wind-fields are incorporated, as evidenced by the average decrease of 15.66% in the MSE, the average decrease of 6.46% in the MAE and the evident decline in the MRE obtained for the IDWS estimation compared to the IDWE estimation (Table 1). The spatial distributions of the relative errors are presented in Table 2.

**Table 1:** Comparison of the interpolation accuracies achieved using MSE, MAE and MRE based on a cross-validation analysis.

Date	PM_2.5_( 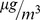 )	wind speed ( 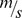 )	wind direction()	MSE of IDWS	MSE of IDWE	MAE of IDWS	MAE of IDWE	MRE of IDWS	MRE of IDWE	Improvement in MSE (%)	Improvement in MAE (%)
4^th^ May	92.2	2.19	206.2	49.33	62.51	5.81	6.24	−0.36%	−1.21%	21.07%	7.40%
18^th^ May	118.8	1.96	194.2	105.86	129.71	8.69	8.91	0.93%	2.53%	18.39%	2.53%
21^st^ May	110.8	1.65	93.4	695.81	781.08	18.54	20.99	1.48%	5.03%	10.92%	13.21%
26^th^ May	106.9	1.71	101.9	85.78	90.23	8.17	8.09	−0.27%	0.41%	4.93%	−0.98%
28^th^ May	17.7	1.99	285.3	96.71	113.19	8.22	9.09	0.22%	0.35%	14.56%	10.58%
29^th^ May	78.8	1.53	251.7	13.83	18.22	2.82	2.99	−1.63%	−2.59%	24.11%	6.03%

**Table 2:** Distributions of the relative errors of the two methods for the daily PM_2.5_ estimation on 21st May.

PM station Number	Estimation of IDWS	Estimation of IDWE	Measured value	Relative error of IDWS	Relative error of IDWE
A	100.9	100.2	129	−21.78%	−22.33%
B	116.8	115.9	100	16.80%	15.90%
C	115.7	121.6	104	11.25%	16.92%
D	113.7	112.6	95	19.68%	18.53%
E	100.7	106.1	104	−3.17%	2.02%
F	106.2	107.6	98	8.37%	9.80%
G	101.3	103.2	179	−43.41%	−42.35%
H	123.8	136.3	98	26.33%	39.08%
I	111.2	117.5	112	−0.71%	4.91%
J	111.9	113.6	118	−5.17%	−3.73%
K	111	118.4	100	11.00%	18.40%
L	105.6	112.5	91	16.04%	23.63%
M	94.9	95.6	113	−16.02%	−15.40%

**Figure 4:**
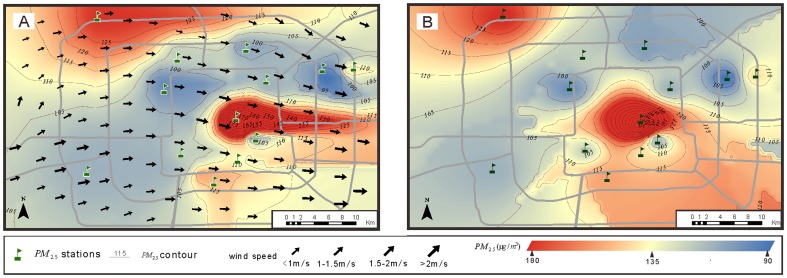
Comparison between the daily PM_2.5_ (May 21^st^) estimations obtained using IDW based on the SWPD and the Euclidean distance. (A)Interpolation results obtained using IDWS. (B) Interpolation results obtained using IDWE.

In addition to the benefit of lowering these three statistics, improvements are evident when the estimation surfaces obtained using two methods are compared visually (Fig. 4). The two methods produce different distributions when the PM_2.5_ value measured at a single location is much higher than those measured at surrounding stations. In the results obtained using IDWS, greater continuity is apparent on the downwind side of the downtown area and there is a shorter dispersion distance on the upwind side, as would be expected from the wind-flow effect (Fig. 4A). IDWE methods always produce an eye-shaped pattern in such cases, which is commonly considered to be a major shortcoming of this interpolation method (Fig. 4B). Under the complex local wind-field northwest of the urban area, the results of the interpolation method proposed in this paper also exhibit an accordingly complex anisotropy (Fig. 4A). However, the estimation surface obtained using IDWE fails to capture this feature (Fig. 4B).

### Interpolation of the hourly PM_2.5_ concentration

**Figure 5:**
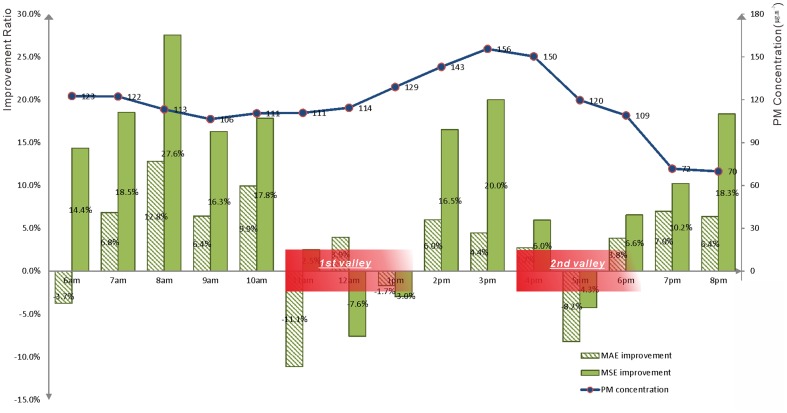
Temporal variation in the hourly PM_2.5_ concentration and the improvement ratio of IDWS with respect to IDWE on May 26^th^.

Here, we consider the process of interpolating the PM_2.5_ concentration on a smaller temporal scale. Although IDWS outperforms IDWE on most experimental dates, the improvement in the MSE on the 26^th^ of May is only 4.93%, and the MAE of IDWS is larger than that of IDWE (Table 1). On May 26^th^, the PM_2.5_ concentration increased gradually during the diurnal hours, reached a peak at approximately 4 pm and then decreased dramatically because of the washout caused by a moderate rainfall event that offset the impact of increasing traffic volume during the evening rush hour (Fig. 5). The hourly measured PM_2.5_ concentrations were interpolated from 6 am to 8 pm. Twelve of the 15 experimental hours exhibit smaller MSE values in the IDWS estimation than in the IDWE estimation, whereas the remaining three hours exhibit larger MSE values, suggesting that the incorporation of the wind-fields had a negative influence on the interpolation accuracy during these three hours. The hours immediately preceding and immediately following these three hours also exhibit a limited improvement of less than 10%. Moreover, the improvement-ratio curve of the MAE follows a similar trend. Thus, two valleys appear in the curves: at noon and at sunset.

**Figure 6:**
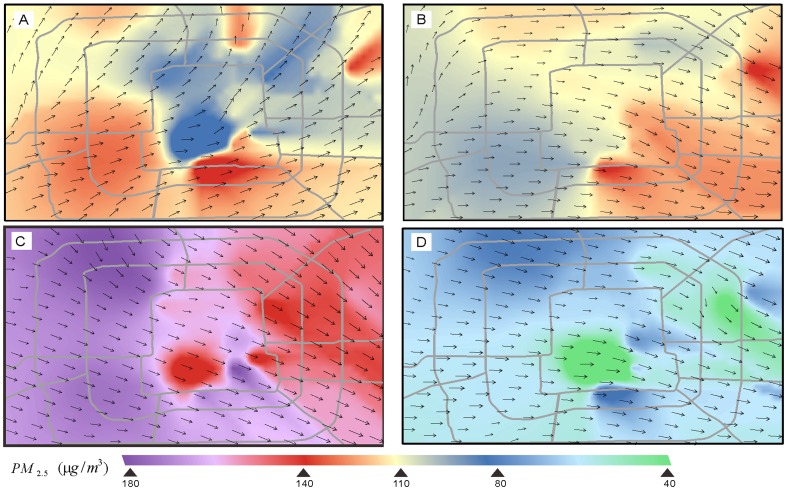
Interpolation results of the hourly PM_2.5_ concentrations on May 26^th^ at various times: (A) 8 am, (B) 12 pm, (C) 4 pm and (D) 8 pm.

The prevailing wind-direction was approximately 65° NE in the morning (Fig. 6A) and changed to 120° SE in the afternoon (Fig. 6C). From 11 am to 1 pm, corresponding to the first valley in both improvement-ratio curves, the prevailing direction experienced dramatic variations from northeast to southeast (Fig. 6B), which limited the accuracy of the wind-field measurements. Because modest direction errors on the order of 10 degrees can lead to large errors in the estimation of air-pollution trajectories, the effectiveness of the proposed methodology no longer holds in cases of strongly varying wind-direction [36]. As is also indicated by the meteorological data set, the study area experienced rainfall from 4 pm to 6 pm, corresponding to the second valley. Because precipitation accelerates the deposition of particulate matter, the transport effects of the wind-field were reduced (Fig. 6D). However, under weather conditions with fewer or weaker variations, the methodology proposed in this paper yielded better results on an hourly temporal scale than did the conventional interpolation method.

## Discussion

This study demonstrates the potential of incorporating wind-field into interpolation using the IDWS approach. In addition to minimizing estimation errors, a major advantage of this approach stems from its ability to reproduce complex nonlinear features caused by the wind-flow effect. This capability deserves further investigation for its potential use in studies of air-pollution and the negative health effects thereof. As shown in Fig. 4A, the asymmetric distribution of PM_2.5_ on the two sides of the downtown area suggests that residents living east of downtown were exposed to higher concentrations, whereas those living to the west were protected by the wind. By contrast, the symmetric distribution predicted by IDWE (Fig. 4B) may overestimate the PM_2.5_ exposure of upwind residents. Furthermore, the estimation surface obtained using IDWS exhibits greater downwind continuity. Ignoring the wind-flow effect will lead to the underestimation of downwind dispersion distance and the overestimation of dispersion distances in other directions. The IDWS technique also enables the modeling of smaller-scale variations, which can reduce prediction uncertainties in exposure assessments.

As demonstrated in the previous sections, the proposed method produced relatively inaccurate estimates on certain experimental dates. A combination of the dramatically changing wind-direction at noon and the precipitation that occurred at sunset led to poor performance on May 26^th^. The daily PM_2.5_ on May 20^th^ was also interpolated, and the improvement in the MSE with respect to the results obtained using IDWE was only 3.31%. Although the daily average wind-speed was greater than 1.5 m/s on May 20^th^, the major pollutant was dust particles caused by blowing sand, which was assumed to be the major source of the prediction uncertainty. These results suggest the need for careful evaluation of the specific weather conditions prior to including the wind-field using IDWS.

The basic version of IDW was applied to test the feasibility of the novel distance metric proposed in this study. Neither the problem of the influence radius nor the problem of zero distance was considered. Some variants of the classic method may be used to verify the validity of the SWPD or even to achieve more accurate estimations. Although it offers a number of advantages, IDW always achieves poorer performance than kriging or other more sophisticated interpolation methods. Thus, now that the effectiveness of the SWPD has been demonstrated and the metric has been shown to offer improved accuracy and realistic visual representation, a need exists to combine this distance metric with a more robust technique to obtain prediction surfaces with higher accuracy [23].

The incorporation of secondary information, instead of relying solely on station measurements, enables the estimation surfaces obtained to reflect localized variations and thus improves the predictive capacity of the analysis [34]. Most currently used auxiliary data are scalar, and little weight is given to vector data. One objective of this paper is to propose a methodology for incorporating vector-type secondary information into interpolation. Now that the feasibility of this methodology has been confirmed, a more general method that is capable of including both scalar-type data and vector-type data is desirable.

The primary intent of this study was to verify the effectiveness of the proposed method, so little attention was directed toward improving the computing efficiency. All algorithms were implemented using C++ with no optimization, and the visualization was performed using ArcGIS (ESRI). At the current stage of development, the time required to generate one estimation surface for the study area is approximately 10 minutes. Further research is necessary to accelerate the calculation and to allow this technique to be used for real-time estimation.

The results of cross-validation and visual assessment have demonstrated that including wind-field in the interpolation of the PM_2.5_ concentration improves the predictive performance. However, the experiments were conducted only in the core area of Beijing during six selected days in May. The method should be assessed over much longer monitoring periods spanning all four seasons to confirm its year-round effectiveness. Additional measurements are also required to confirm the usefulness of the SWPD or even to discover a better distance metric. The interpolation of other types of air-pollution, such as nitrogen dioxide and coarse particulate matter, should also be performed to verify that the model has general applicability. Furthermore, wind-fields with higher resolution may have the potential to improve the predictive capability of the technique and deserve further research.

## Conclusions

Three major conclusions can be drawn from this study:

1. Wind-fields are of great importance to studies of the negative effects of airborne pollutants. Incorporating wind-fields into the spatial interpolation of air-pollution distributions serves to enhance the predictive capability of such interpolation.
2. The shortest wind-field path distance (SWPD) shows great potential for determining the spatial dependence and enables SWPD-based interpolations to capture complex features of air-pollution distributions with higher accuracy than methods based on the Euclidean distance.
3. The workflow proposed in this paper, which consists of wind-field generation, shortest-path analysis and IDW in conjunction with the SWPD, has been demonstrated to be a robust technique for predicting urban-scale PM_2.5_ concentrations.

## Supporting Information

Movie S1 **The spatial-temporal variance of PM_2.5_ concentrations obtained by IDWE in the study area on May 26^th^.**
(WMV)
